# A Conjoint Analysis of Consumer Preferences on Shiitake Mushrooms: A Case Study of the Republic of Korea

**DOI:** 10.3390/foods15020217

**Published:** 2026-01-08

**Authors:** Changjun Lee, Kidong Kim

**Affiliations:** Division of Forest Strategy Research, National Institute of Forest Science, Seoul 02455, Republic of Korea

**Keywords:** conjoint analysis, consumer preference, food marketing strategy, non-timber forest product (NTFP), shiitake mushrooms

## Abstract

Shiitake mushrooms (*Lentinula edodes*) are widely consumed as a key health food in the Republic of Korea. However, they face declining production value and consumption, necessitating a shift from production-focused research to an understanding of consumer demand. The aim of this study was to quantify Korean consumers’ trade-offs among key shiitake attributes and to derive actionable marketing strategies to expand domestic consumption. We conducted an online survey (*n* = 500) to quantify consumer utility for four key attributes: cap size (two levels), cap color (two levels), origin (two levels: domestic (Korean) and imported (Chinese)), and price (four levels per 500 g). The results identified price as the most important attribute (relative importance = 46.41%), followed by origin (19.85%), cap color (17.10%), and cap size (16.64%). Utility analysis (part-worths) revealed a distinct dual preference: consumers value both low-priced shiitake (KRW 4000 (USD 2.9)/500 g) for personal consumption and high-priced options (KRW 13,000 (USD 9.5)/500 g) for gifting. Consumers showed a clear preference for dark-colored caps, while the aggregate-level utility difference between origin levels was small. A Logit model simulation indicated the highest predicted shares for profiles priced at KRW 13,000 (15.9%) and KRW 4000 (15.7%), consistent with a polarized value–premium structure. These findings indicate that Korean producers should adopt a dual strategy: developing low-cost products to stimulate general consumption while simultaneously marketing high-quality, dark-colored, domestically produced shiitake as premium gift items, thereby establishing effective food choice strategies in a competitive market. Although the empirical setting is the Republic of Korea (with ‘Chinese’ included only as an imported-origin level representing the main foreign competitor), the findings speak to broader specialty-food contexts where import competition and dual-purpose purchasing (everyday use vs. gifting) shape attribute trade-offs.

## 1. Introduction

Shiitake mushrooms (*Lentinula edodes*) are widely cultivated and consumed worldwide [1]. While they are a major non-timber forest product (NTFP) in the Republic of Korea, shiitake mushrooms (*Lentinula edodes*) are not only an indispensable traditional ingredient in Korean food culture but also a representative health food known for their various benefits, such as strengthening immunity and preventing adult-onset diseases [1,2]. Furthermore, NTFPs are widely recognized as a critical source of cash income for forest households, playing a vital role in rural economies [3,4]. Despite this positive perception, shiitake mushrooms face competition from relatively low-priced alternatives, such as king oyster mushrooms (*Pleurotus eryngii*) and oyster mushrooms (*Pleurotus ostreatus*) [5]. Furthermore, the primary consumer base for shiitake is concentrated among older demographics, while among the younger generation, who consume a wider variety of food ingredients, consumption remains relatively low [6]. In this situation, the traditional supplier-centric market approach, which focuses on improving production technology, has limitations. In the current market, where consumer choice is paramount, a deep understanding of demand is essential [7,8]. Identifying how consumers make final decisions by weighing various attributes at the point of purchase (such as price, size, freshness, and origin) is crucial for expanding the market [9,10].

Previous research on shiitake mushrooms has predominantly focused on production aspects, such as cultivation techniques, pest management, and nutritional analysis, with relatively few efforts to deeply understand consumers’ changing demands [11]. In today’s market, where consumer choice is enhanced, achieving sustainable consumption is difficult without an in-depth analysis of demand. While promoting the excellent benefits of shiitake mushrooms is important, it is necessary to understand quantitatively how consumers evaluate various attributes (such as price, origin, cap size, and color) and how the interplay between these attributes influences their selection. The focal attributes (cap size, cap color, origin, and price) were selected through a two-step process: a review of prior studies on mushroom purchasing behavior and consultations with shiitake wholesalers to ensure market relevance. Specifically, the Chinese-origin level is operationalized as the primary imported competitor in the Korean retail market; accordingly, the analysis focuses on Korean consumers. Further details on attribute selection are provided in Section 2.3.

Methodologically, conjoint analysis and discrete choice experiments have been extensively employed in food consumer research to decipher the trade-offs consumers make between product attributes. Recent studies published in Foods have successfully applied these methodologies to a wide range of products. For instance, Srisukwatanachai et al. conducted a comparative choice experiment in China and Thailand to examine sensory and country-of-origin attributes for rice [12], and Jordaan et al. analyzed how intrinsic and extrinsic cues shape perceived value in a South African consumer sample for pork products [13]. Furthermore, Go and Yi demonstrated the applicability of these quantitative methods specifically within the Korean market, estimating consumer valuation for sustainable seafood [14]. These studies collectively suggest that understanding the trade-offs consumers make between attributes—such as price, origin, and visual characteristics—is key to market success. Building on this established framework, this study applies conjoint analysis to the specific context of Korean shiitake mushrooms.

The purpose of this study is to propose empirical strategies for expanding the domestic shiitake mushroom market by closely analyzing consumer consumption patterns and preferred attributes. To achieve this, we identified the key attributes influencing consumers’ shiitake mushroom purchase decisions and evaluated the relative importance and preference level for each attribute. Based on the analysis results, we simulated market share predictions and proposed directions for consumption expansion strategies.

To accomplish the research objectives, we employed conjoint analysis and conducted a choice simulation to examine implicit trade-offs consumers make at the point of purchase [15,16]. Detailed methodological procedures are described in Section 2.

This study examines how Korean consumers trade off key shiitake attributes at the point of purchase and assesses whether the price structure implies distinct market positioning for everyday consumption versus gifting. The empirical results and implications are presented in Section 3 and Section 4. This paper is structured as follows: Section 2 details the materials and methods, including the survey design and conjoint analysis; Section 3 presents the key findings; Section 4 discusses the implications of these results; and Section 5 provides the conclusions.

## 2. Materials and Methods

### 2.1. Survey Overview

To measure the relative importance of shiitake mushroom attributes for the conjoint analysis and to investigate consumer purchasing behavior, an online survey was administered to 500 Korean nationals over four days, from 10 to 13 September 2024. The survey was administered by a professional market research firm (Research Lim, Seoul, Republic of Korea) using its proprietary online survey system. In addition to the eligibility screening question and mandatory response fields, strict quality-control procedures were implemented to ensure data integrity. These included duplicate-response prevention (via unique respondent IDs) and logic trap questions to identify inattentive respondents. Responses failing these quality checks were automatically filtered by the firm prior to the delivery of the final dataset; therefore, the analytic sample consists of 500 quality-screened, valid responses. In this study, “shiitake mushroom” referred to fresh shiitake, while dried shiitake was explicitly described as “dried shiitake.” The questionnaire included items on shiitake purchase volume, purchase frequency, information acquisition channels for purchases, primary packaging type, main purchase location, reasons for using the main purchase location, explanations and photos to enhance the understanding of shiitake attributes and levels, and respondent characteristics. All shiitake attributes and their respective levels were presented with illustrations and photographs to ensure that respondents fully understood them before participating in the survey.

The preliminary questionnaire was developed based on previous studies regarding consumer preferences for mushrooms [17,18] and conjoint analysis methodology [19,20]. To ensure content validity, the draft underwent a rigorous review by internal experts at the National Institute of Forest Science (NIFoS). Subsequently, a pre-test was conducted with seven individuals with prior shiitake consumption experience to verify the clarity and readability of the survey items.

The target population for this study was adults who had purchased shiitake mushrooms within the one-year period of 2023. To reduce potential sample distortion and improve alignment with the population distribution, quota sampling was employed based on the Resident Registration Demographic Statistics of Korea. Specifically, the sample was proportionally allocated according to gender and residential region. However, age was not applied as a quota variable because the study aimed to capture the natural demographic profile of recent shiitake purchasers, which prior evidence suggests is concentrated among older consumers in the Republic of Korea [6,17]. The final valid sample size was established at 500, which exceeds the minimum sample size required for conjoint analysis [21], and the survey was administered online. Eligibility was restricted to adults who had purchased fresh shiitake mushrooms within the previous year (i.e., during 2023) to ensure a recent purchase experience and reduce recall bias. In this study, ‘purchased’ refers to respondents who personally made the purchase decision and completed the transaction, ensuring they were the primary shoppers. To minimize misclassification, the survey clearly defined ‘fresh shiitake’ (vs. dried shiitake) and provided reference photographs before screening. Regarding compensation, participants received a small, fixed incentive (e.g., coupons) in accordance with the market research firm’s standard policy; this incentive was uniform and not contingent on specific response patterns.

### 2.2. Conjoint Analysis

Conjoint analysis is a multivariate analysis technique, introduced to the marketing field by Green and Rao (1971), based on the premise that consumers make decisions by comprehensively considering multiple attributes when choosing a product or service [19,22]. The purpose of this analysis is to estimate part-worths by measuring the relative importance consumers assign to a product’s various attributes and the utility of each attribute level [23,24]. To this end, it mimics a realistic purchase decision-making process by presenting consumers with hypothetical product profiles created by combining levels of various attributes and having them evaluate their preferences [25,26].

Using the estimated part-worths, the market share of virtual products with different attribute combinations can be predicted. Market share prediction is analyzed by applying a specific choice rule. Common models include the deterministic Maximum Utility model and probabilistic BTL (Bradley–Terry–Luce) and Logit models [27,28]. The former assumes that each consumer chooses only the one alternative with the highest utility, whereas the latter models assume that a higher utility increases the probability of selection. The Logit model, in particular, assumes that the utility of an alternative consists of a deterministic component (derived from the attribute levels) and a random error component. Based on McFadden’s (1972) Random Utility Theory [29], the probability that a consumer chooses a specific alternative is calculated as the ratio of the exponential of the utility of that alternative to the sum of the exponentials of the utilities of all available alternatives ( $P_{i}=e^{U_{i}}/\sum e^{U_{j}}$). This approach is widely used for more realistic market predictions as it reflects the uncertainty inherent in consumer choice. Indeed, previous studies using choice simulations have shown that strategies emphasizing key attributes, such as ‘origin’, can be leveraged to gain a competitive advantage and increase market shares [27]. Such simulations provide a useful scientific basis for launching new products or establishing related policies [30]. It is worth noting that while the attributes used in this study (e.g., size, color, origin) are commonly discussed in commodity science as quality cues for product characterization and quality evaluation, conjoint analysis serves a distinct analytic purpose. Rather than defining objective quality standards, this method estimates consumers’ part-worth utilities (preference weights) for each attribute level by observing how they trade off multiple cues simultaneously. In this sense, conjoint analysis complements commodity science by quantifying market-relevant preferences and trade-offs among familiar quality cues.

### 2.3. Measurement Instrument

The attributes for the conjoint analysis—cap size, cap color, origin, and price—were selected through a systematic two-step process. First, potential influencing factors were derived from a review of prior literature on mushroom consumption behavior [17,18]. Second, to ensure market suitability and practical relevance, these factors were verified and finalized through in-depth consultations with shiitake wholesalers. This process confirmed that these four attributes represent the most fundamental criteria consumers use at the point of purchase, minimizing the risk of overlooking other influential factors. The origin attribute was included as an important policy element in accordance with recent shiitake labeling guidelines, as in the Republic of Korea, origin is generally distinguished between ‘domestic’ (Korean) and ‘Chinese’ (Table 1). Recent work has also proposed analytical approaches for origin identification to support labeling integrity in shiitake markets [31]. Specifically, the attribute levels were determined based on empirical data and expert consultations to ensure market realism. The price levels (KRW 4000 to 13,000) were derived from the internal data of the ‘Survey on Consumption Behavior of NTFPs,’ conducted annually by the NIFoS targeting 1100 consumers. These price ranges were further validated through in-depth consultations with mushroom wholesalers to reflect actual retail conditions. Regarding cap size and color, these attributes were selected as key visual cues easily identifiable by consumers, distinct from technical grading criteria (such as cap thickness or cap opening). These visual standards were finalized through face-to-face interviews with both producers and wholesalers. Regarding cap color, to ensure that respondent preferences were driven solely by color differences rather than morphological variations, standardized reference images were utilized. Specifically, a single baseline image of a shiitake mushroom was digitally processed to create two distinct levels: ‘light’ (representing a bright, beige tone) and ‘dark’ (representing a deep brown tone). These images were presented side-by-side to minimize potential confounding effects from differences in shape or texture. Regarding other search attributes, although ‘packaging type’ and ‘certifications (e.g., Organic, Good Agricultural Practices [GAP])’ were identified as relevant factors in our preliminary survey (detailed later in Section 3.3), they were excluded from the final conjoint design for strategic reasons. Since shiitake mushrooms are retailed in diverse formats ranging from bulk to various small packages depending on the sales channel, including packaging as a variable would have excessively increased the complexity of the experimental design. Therefore, to minimize respondent cognitive burden and ensure robust data, this study prioritized the four most critical attributes—price, origin, cap size, and color—that allow consumers to intuitively judge the intrinsic quality of the mushroom itself.

The levels for the four attributes were set as follows: two levels for cap size, two for cap color, two for origin, and four for price. Evaluating part-worths using the full profile method would generate a total of 32 profiles (2 × 2 × 2 × 4). However, it is practically impossible for respondents to consistently rank all 32 profiles, which could lead to respondent fatigue and a decline in response quality [21,32]. Therefore, to compensate for the drawbacks of the full profile method, in this study, we applied a fractional factorial design. This method allows for the estimation of main effects while assuming that interaction effects between attributes are negligible, thereby significantly reducing the number of profiles required for evaluation [20]. We prioritized estimating main effects to minimize respondent cognitive burden during the ranking task. While we acknowledge that interaction effects (e.g., price × origin) may exist and that assuming their negligibility presents a limitation, this design was chosen to ensure data quality by preventing respondent fatigue. Future studies could employ larger choice-based conjoint (CBC) designs to explicitly test these interactions.

The profiles were generated using the orthogonal design procedure (ORTHOPLAN) in SPSS V26.0. This algorithm generates a subset of profiles where the attributes are statistically independent (orthogonal) to one another, ensuring that the estimate of each attribute’s utility is not confounded by others. From the thirty-two total possible combinations, this procedure derived eight optimal profiles as the minimum set necessary to estimate the main effects with sufficient statistical power (Table 2) [15]. Respondents evaluated their preferences for the eight derived profiles from most to least preferred using the rank-order method [33].

The collected preference rank data were used to estimate the part-worth values for each attribute level and the relative importance of each attribute through conjoint analysis. Based on the estimated part-worths, the market shares of the eight profiles in a virtual market were predicted using a choice simulation method. Specifically, the total utility for each profile was calculated by summing the part-worths of its constituent attribute levels. These total utility values were then converted into predicted market shares by applying a probabilistic choice rule. While various models exist for market share prediction, this study applied the Logit model, which is frequently employed for predicting the market share of non-durable, higher-priced goods.

### 2.4. Statistical Analysis

Data analysis was performed using SPSS Statistics V26.0. The study employed a fractional factorial design and a Logit model to estimate attribute part-worths and predict market shares. The goodness of fit for the conjoint model was assessed using Pearson’s *R* and Kendall’s tau statistics. Additionally, to identify significant differences in consumer purchasing behavior based on demographic characteristics (age, gender, and income), cross-tabulation analyses and Pearson’s Chi-square tests were conducted. Statistical significance was established at *p* < 0.05.

## 3. Results

### 3.1. Sociodemographic Characteristics

To ensure data quality, the online survey included a screening question that restricted participation to consumers who had purchased shiitake mushrooms in 2023. Furthermore, systematic constraints (e.g., mandatory response fields) were implemented within the survey platform to prevent incomplete or unfaithful responses. Consequently, a total of 500 valid responses were collected and used for the final analysis without the need for post hoc exclusion. The sociodemographic characteristics of the respondents are summarized in Table 3.

Analysis of the sociodemographic characteristics showed that males accounted for 49.6% (*n* = 248) and females for 50.4% (*n* = 252). In terms of age distribution, those in their 60s were the largest group at 32.2% (*n* = 161), followed by those in their 50s (20.0%, *n* = 100) and 40s (18.2%, *n* = 91). Although the age distribution appears older than that of the general Korean population, this study did not aim to represent the population at large; eligibility was restricted to adults who had purchased shiitake mushrooms during 2023. Given that shiitake purchasing in the Republic of Korea is commonly concentrated among older cohorts [6,17], the observed age profile is consistent with the characteristics of the active purchaser population and should be interpreted within that scope. Regarding income level, the highest proportion of respondents (17.2%, *n* = 86) had an average monthly household income in the KRW 3 million range, followed by the KRW 4 million (15.8%, *n* = 79) and KRW 5 million ranges (13.2%, *n* = 66). For family size, respondents with two to three family members constituted 60.4% (*n* = 302) of the total, followed by those with four or more members (30.4%, *n* = 152) and single-person households (9.2%, *n* = 46). Regarding residence, 28.6% (*n* = 143) lived in Seoul and 19.0% (*n* = 95) in Gyeonggi-do, with residents of the Seoul metropolitan area (Seoul, Gyeonggi) accounting for 47.6% (*n* = 238) of the total.

### 3.2. Purchasing Behavior

As summarized in Table 4, regarding annual purchase frequency, 26.4% (*n* = 132) of respondents purchased shiitake mushrooms “More than 6 times” a year, followed by those who purchased them “2 times” (24.2%, *n* = 121) and “1 time” (20.8%, *n* = 104). In terms of average purchase volume per trip, the largest proportion of respondents purchased “Less than 300 g” (36.8%, *n* = 184), followed by “301 g–500 g” (29.0%, *n* = 145). Only 8.4% (*n* = 42) purchased “More than 1 kg”. Notably, many respondents reported purchasing shiitake only once or twice per year, indicating that the market includes many infrequent purchasers. This does not imply an inability to evaluate products, as the conjoint task focuses on observable search cues (price, origin, and visual appearance) presented with standardized images. Rather, the prevalence of infrequent purchasing underscores the importance of demand-side strategies aimed at converting occasional buyers into regular consumers.

Regarding reasons for purchasing fresh (vs. dried) shiitake, “To consume them fresh” was the most common reason cited (46.2%, *n* = 231), followed by “For various culinary uses” (31.6%, *n* = 158) and “Lower price (compared to dried)” (11.4%, *n* = 57). “Better taste (compared to dried)” was selected by 10.0% (*n* = 50). Other opinions included factors such as time-saving and convenience.

For information acquisition channels, “Family, friends, relatives, neighbors” was the highest at 35.6% (*n* = 178), followed by “Store/sales staff” (22.6%, *n* = 113) and “Internet portal” (15.2%, *n* = 76).

The most common purchase location was “Offline hypermarket (large-scale mart)” (30.6%, *n* = 153), followed by “Traditional market” (19.4%, *n* = 97), “Corporate supermarket” (17.0%, *n* = 85), and “Local supermarket (non-corporate)” (12.4%, *n* = 62). The primary reasons for using the purchase location were “Easy access” (34.2%, *n* = 171), followed by “Good quality” (21.4%, *n* = 107) and “Low price” (21.0%, *n* = 105).

To examine the relationship between demographic characteristics and purchasing behavior, a cross-tabulation analysis (Chi-square test) was conducted regarding age, gender, and income (Table 5). The results revealed specific patterns of significance. First, gender was a significant factor influencing purchase frequency (*p* < 0.001); female respondents showed a higher proportion of heavy users (31.0%) compared to males (21.8%). Second, age significantly influenced information acquisition channels (*p* < 0.001) and purchasing locations (*p* < 0.05). Younger consumers (20s) relied more on online channels (e.g., YouTube 21.7%) and corporate supermarkets (27.5%), whereas older consumers (60s+) depended heavily on store staff (34.2%) and traditional markets (19.3%). However, income level did not show statistically significant differences across any purchasing behaviors (*p* > 0.05), suggesting that shiitake mushroom consumption is relatively independent of household income.

### 3.3. Considerations for Shiitake Mushroom Purchases

Respondents were asked to rate their considerations when purchasing shiitake mushrooms on a 5-point Likert scale, with options including taste, hygiene and safety, regional specialty status, origin, preparation convenience, packaging condition, variety, and national certification related to food safety.

As shown in Table 6, the analysis of 500 responses showed that “hygiene and safety” and “origin” were paramount considerations, with mean scores above 4.0 on the 5-point Likert scale. Other factors (including taste, regional specialty status, convenience, packaging, variety, and national certification) all had mean scores in the 3.0 range, indicating a “moderate” level of consideration.

It is important to clarify that the exclusion of hygiene/safety and taste from the conjoint task does not imply that these factors are unimportant; rather, it reflects a deliberate design choice. In online experiments, ‘taste’ is an experience attribute that cannot be objectively evaluated via visual cues, and ‘hygiene/safety’ typically functions as a non-compensatory baseline prerequisite (i.e., consumers assume a minimum safety standard) rather than a tradable attribute, consistent with a two-stage decision process [34]. Therefore, to avoid unrealistic trade-offs and measurement bias, the conjoint design focused strictly on ‘search attributes’ (price, origin, appearance) that consumers can directly assess at the point of purchase. Future research could integrate sensory evaluation protocols or credence cues (e.g., certification labels) within expanded choice-based designs to jointly model these drivers [35].

### 3.4. Estimation of Importance by Attribute and Part-Worths

As detailed in Table 7, the conjoint analysis results showed that price was the most important attribute (46.41%), followed by origin (19.85%), cap color (17.10%), and cap size (16.64%).

An analysis of the part-worth (utility) values for each attribute revealed that for the price attribute, “KRW 13,000” had the highest part-worth value, followed by “KRW 4000”. The reason for the high part-worth of the former is that shiitake mushrooms are often sold as high-end gifts in the Republic of Korea; thus, consumers associate a price of KRW 13,000 or more with high value. While it is common in conjoint analysis to set price as a linear variable (assuming utility decreases as price increases), this study set price as a discrete (nominal) variable, because the Korean shiitake market is distinctly segmented into premium and regular markets. This linear setting would have incorrectly yielded a result where utility increased with price. Notably, the part-worth estimates for price exhibit a clear non-linear (U-shaped) pattern (Table 7): both the lowest price (KRW 4000) and the highest price (KRW 13,000) yield positive utilities, whereas the intermediate prices (KRW 7000 and 10,000) yield negative utilities. Interpreted ceteris paribus, this implies that the mid-price range is not naturally perceived as an attractive compromise. Rather, it may be viewed as neither sufficiently economical for routine household consumption nor sufficiently ‘premium’ to function as a strong quality or gifting signal. This pattern supports the presence of a structurally segmented market and provides a concrete rationale for treating price as a categorical (nominal) attribute rather than imposing a monotonic linear price–utility relationship.

For origin, the aggregate-level part-worth difference between the two levels was very small (Table 7), suggesting no clear directional dominance in the averaged model; however, the relatively high importance score indicates that origin remains a salient cue and may reflect heterogeneous valuations across consumers. For cap color, “dark color” was preferred (higher part-worth) over “light color”. Finally, for cap size, “8 cm or more” had a higher part-worth value than “less than 8 cm”.

### 3.5. Market Share Prediction Using Simulation

A simulation method (Logit model) was applied to predict the potential market share for the eight virtual profiles used in the conjoint analysis. As shown in Table 8, the results predicted the highest market share (15.9%) for Profile 4, which was “Chinese”, “dark-colored”, “less than 8 cm” in cap size, and priced at “13,000 KRW/500 g”. This was closely followed by Profile 3 (“Korean”, “dark-colored”, “8 cm or more”, and priced at “4000 KRW/500 g”) with a 15.7% market share. Notably, the top-ranked profiles (Rank 1 to 4) all featured “dark color”, suggesting that cap color operates as an important visual cue alongside price. In the Logit model, the total deterministic utility of each profile is calculated as the sum of its part-worths across all attribute levels, and the predicted choice probability (market share) is proportional to the exponential of that total utility, consistent with the Random Utility Theory [29]. Accordingly, the positive utility of “dark color” combined with preferred price points (low or high) can outweigh the relatively small utility difference between origin levels in the aggregate model.

As indicated by the attribute importance results, price was determined to be the most significant factor influencing market share. At the same time, the simulation suggests that cap color may serve as a practically meaningful visual cue that can reinforce market positioning when paired with appropriate price strategies.

## 4. Discussion

The decline in production value of fresh shiitake mushrooms, a major NTFP in the Republic of Korea, underscores the urgent need for market strategies based on consumer demand. This study’s analysis of purchasing behavior reveals a polarized market. A significant segment of consumers (45.0%) purchases shiitake infrequently (twice a year or less), while another segment purchases them frequently (six or more times). This polarization and the overall stagnation in consumption frequency align with findings by Kim et al. [5], who attributed the slowdown in shiitake consumption to economic downturns and the increasing supply of lower-priced substitute mushrooms, such as king oyster and enoki mushrooms. Additionally, the high importance placed on hygiene/safety and origin (Table 6) may be consistent with increasingly safety- and quality-oriented food consumption trends in the Republic of Korea, where consumers often rely on credence cues to infer product value.

Our findings also sketch a portrait of the primary consumer: they are often of an older demographic and rely on offline information sources. This demographic trend is consistent with Min et al. [6], who reported that shiitake mushrooms are preferred by older age groups (50s and 60s) and high-income households, while consumption among younger generations remains relatively low. This consumer profile, combined with their preference for offline hypermarkets due to “easy access,” helps to explain why price emerged as the most critical purchasing attribute (46.41%), and it indicates that the present implications primarily inform strategies for the current purchaser base rather than a complete blueprint for converting younger non-purchasers.

The central finding of this study is the distinct dual preference regarding price: consumers valued both low prices (4000 KRW/500 g) and high prices (13,000 KRW/500 g). This strong emphasis on price (46.41%) contrasts evidence from a prior study of consumers in China reported by Min and Koo [18], who prioritize quality attributes such as freshness and safety over price. We interpret this result as evidence of a unique bimodal market structure in the Republic of Korea: a “general consumption market” driven by price sensitivity and a “premium gift market” where high price signals quality. An additional implication of the price results is the meaning of the negative utilities for the mid-price levels (KRW 7000 and 10,000/500 g). These negative part-worths suggest a ‘missing middle’ in price-based positioning: mid-priced shiitake options may be perceived as overpriced relative to the low-price benchmark relevant for everyday consumption (especially under competition from cheaper substitute mushrooms), while also being insufficiently expensive to convey the prestige or quality signal expected in gift-oriented purchases [36,37]. Practically, this indicates that a mid-price strategy is likely to be vulnerable unless it is paired with clearly differentiating cues that justify the price (e.g., stronger quality signaling and communication through branding and presentation, or a bundle of favorable attribute levels such as domestic origin and preferred appearance). This interpretation is also consistent with the simulation results (Table 8): profiles priced at KRW 10,000/500 g can still achieve strong predicted shares when combined with high-utility non-price attributes, implying that mid-priced products may require ‘compensating’ strengths to overcome the baseline penalty associated with the mid-price level. While Chinese consumers show lower price sensitivity [18], Korean consumers exhibit high sensitivity for daily consumption due to competition from lower-priced alternatives [5], yet simultaneously value high-priced shiitake as premium gifts. This reflects the cultural context of the Republic of Korea, where high-quality agricultural products are highly valued as gifts, a trend also observed in previous studies [17]. Consequently, a dual market strategy is essential for producers, a recommendation which is strongly supported by the market share simulation (Table 8). The simulation indicates the highest predicted shares for Profile 4 (KRW 13,000; 15.9%) and Profile 3 (KRW 4000; 15.7%), reinforcing a value–premium split rather than a strictly monotonic price effect. The simulation results underscore the dominance of price-driven trade-offs in this market. Because the aggregate-level utility difference between origin levels is small, the Logit model evaluates complete product bundles, meaning that an origin disadvantage can be offset by stronger compensating benefits—most notably preferred price points and preferred visual cues such as dark cap color. This highlights a practical implication for producers: origin alone may be insufficient to win the value segment unless paired with competitive pricing or other strong quality signals, whereas domestic origin can be strategically leveraged in premium or gift-oriented offerings where consumers are less price-sensitive. In terms of international comparability, our findings are directionally consistent with stated-preference and experimental evidence that origin (including “local” labels) and visual/quality cues can carry value premiums, while the role of price and the magnitude of local/origin premiums vary across products, settings, and consumer segments [10,26,36,37,38,39]. However, the Korean case appears more polarized: unlike evidence from China where consumers emphasize quality-related cues over price [18], our estimates show a pronounced U-shaped price–utility pattern consistent with a split between routine household purchases and gift-oriented purchases. Evidence from the U.S. mushroom market also suggests meaningful preference heterogeneity across consumer segments [40], which aligns with our interpretation that aggregate-level origin effects can be muted even when origin remains salient as a cue. Related evidence from an African context also documents non-trivial trade-offs and heterogeneity in mushroom preferences (e.g., Ghana), reinforcing that cue-based valuation mechanisms extend beyond the Republic of Korea and East Asia [41]. While direct quantitative comparison is limited by differences in product definitions and experimental designs, positioning the Korean results within this international stated-preference literature clarifies both the convergent mechanisms (cue-based valuation) and the context-specific divergence (value–premium polarization).

Finally, this study has several limitations that should be considered when interpreting the results. With respect to design and measurement, the conjoint analysis deliberately focused on observable search attributes and therefore excluded subjective sensory attributes (e.g., taste, scent) and credence attributes (e.g., hygiene/safety); as a result, the model cannot capture the full spectrum of choice drivers. In addition, the fractional factorial design prioritized main effects and thus did not allow the explicit estimation of interaction effects between attributes (e.g., price × origin). Regarding scope and generalizability, the empirical setting is geographically restricted to the Republic of Korea (with ‘Chinese’ included only as an origin level representing the main imported competitor), and no systematic cross-country comparison was conducted; accordingly, the dual price preference should be generalized to other markets with caution. Regarding sampling and data collection, eligibility was restricted to recent purchasers, which strengthens within-market relevance but limits inference about non-consumers, adoption barriers, and preference formation among younger cohorts who are underrepresented in the current purchaser base. The sample also skews toward older adults, reflecting the current core consumer base; therefore, the implications should be interpreted primarily as strategies for maintaining and activating existing demand rather than as definitive evidence on how to shift preferences among younger or non-purchasing consumers. However, evidence from systematic reviews indicates that dietary behaviors among older adults can be modified through appropriately designed behavioral interventions, suggesting that demand-side strategies targeted at the current core (older) purchaser base can still be impactful [42]. Moreover, although quota sampling was applied by gender and region, data were collected via a web-based online survey administered through a professional market research firm’s system with a small fixed incentive. The incentive mechanism may introduce self-selection bias. Although duplicate-response prevention (unique respondent IDs) and logic-trap screening were implemented to filter inattentive responses, we cannot fully rule out incentive-related participation motives. The online mode may also underrepresent consumers with lower digital literacy, particularly older consumers who rely more on traditional markets and store staff, which may tilt descriptive patterns for information channels and purchase locations and partially influence estimated utilities. Finally, as with stated-preference methods, responses were elicited in a hypothetical setting and may differ from actual purchasing behavior; future work could therefore incorporate incentive-aligned choice tasks and field-based designs to triangulate stated and revealed preferences [38,39].

To address these limitations, future research could adopt mixed-mode designs (e.g., online surveys combined with intercept or face-to-face surveys at traditional markets or telephone-assisted surveys) and consider post-stratification weighting to improve representativeness. Future studies could also explicitly test interactions using larger Choice-Based Conjoint (CBC) designs and validate stated-preference predictions with revealed-preference evidence (e.g., retail scanner/POS data) or incentive-compatible approaches (e.g., experimental auctions and field experiments). Finally, future work should extend the scope to include non-consumers and an opt-out option to identify adoption barriers and to test whether convenience- or channel-based levers can broaden demand among younger cohorts.

Despite these limitations, this study provides robust empirical evidence on how Korean consumers trade off key shiitake attributes under a systematically controlled experimental design. The validity of the results is supported by strong model fit statistics (Pearson’s R = 0.998), and the findings offer actionable, market-relevant insights—particularly regarding the dual market structure and price-driven trade-offs—that can inform practical strategies for revitalization in the Korean shiitake industry.

## 5. Conclusions

To address the declining production value of shiitake mushrooms and to increase the income of forest households, we analyzed Korean consumer preferences using conjoint analysis. The results of this study confirmed that price (46.41%) was the single most critical factor influencing consumer purchasing decisions, far outweighing origin, cap color, or cap size. The market simulations indicated the highest predicted shares at both KRW 4000 and KRW 13,000 per 500 g, supporting a dual positioning strategy for value-oriented everyday consumption and premium gifting.

However, the high utility observed for premium-priced options (13,000 KRW/500 g) indicates the clear presence of a distinct gift market. Therefore, to revitalize the industry, producers must adopt a dual strategy: (1) developing cost-effective production methods to lower prices for general consumption and (2) simultaneously marketing high-quality, dark-colored, domestically produced shiitake as a premium product. As a popular health food, shiitake mushrooms have potential for increased demand, and the observed value–premium split suggests that pricing can operate simultaneously as a cost factor for everyday use and as a quality signal for gifting in specialty-food markets facing import competition.

## Figures and Tables

**Table 1:** Attributes and levels of shiitake mushrooms used in the conjoint analysis.

Attribute	Level
Cap size	≥8 cm
	<8 cm
Cap color	Dark
	Light
Origin	Korean
	Chinese
Price (500 g)	KRW 4000 (USD 2.9)
	KRW 7000 (USD 5.1)
	KRW 10,000 (USD 7.3)
	KRW 13,000 (USD 9.5)

Note: The 2024 annual average exchange rate (USD 1 = KRW 1368.98; Bank of Korea) was applied. In this study, ‘Chinese’ denotes the imported-origin level (the main imported competitor) rather than a respondent country.

**Table 2:** Virtual products obtained by orthogonal design.

ID	Cap Size	Cap Color	Origin	Price (500 g)
1	≥8 cm	Dark	Chinese	KRW 7000 (USD 5.1)
2	≥8 cm	Light	Korean	KRW 13,000 (USD 9.5)
3	≥8 cm	Dark	Korean	KRW 4000 (USD 2.9)
4	<8 cm	Dark	Chinese	KRW 13,000 (USD 9.5)
5	<8 cm	Dark	Korean	KRW 10,000 (USD 7.3)
6	<8 cm	Light	Chinese	KRW 4000 (USD 2.9)
7	<8 cm	Light	Korean	KRW 7000 (USD 5.1)
8	≥8 cm	Light	Chinese	KRW 10,000 (USD 7.3)

**Table 3:** Sociodemographic characteristics of participants in the conjoint analysis.

Sociodemographic Characteristics		Frequency (*n*)	Percentage (%)
Gender	Male	248	49.6
	Female	252	50.4
Age	20–29	69	13.8
	30–39	76	15.2
	40–49	91	18.2
	50–59	100	20.0
	60–69	161	32.2
	More than 70	3	0.6
Monthly household income	Less than KRW 2 million	24	4.8
	KRW 2–3 million	51	10.2
	KRW 3–4 million	86	17.2
	KRW 4–5 million	79	15.8
	KRW 5–6 million	66	13.2
	KRW 6–7 million	59	11.8
	KRW 7–8 million	36	7.2
	KRW 8–9 million	39	7.8
	KRW 9–10 million	21	4.2
	More than KRW 10 million	39	7.8
Family members (No.)	1 person	46	9.2
	2–3 persons	302	60.4
	More than 4 persons	152	30.4
Residential region	Seoul	143	28.6
	Busan	54	10.8
	Incheon	15	3.0
	Daegu	38	7.6
	Daejeon	25	5.0
	Gwangju	26	5.2
	Ulsan	10	2.0
	Sejong	1	0.2
	Gyeonggi	95	19.0
	Gangwon	5	1.0
	Chungbuk	13	2.6
	Chungnam	15	3.0
	Jeonbuk	13	2.6
	Jeonnam	8	1.6
	Gyeongbuk	8	1.6
	Gyeongnam	23	4.6
	Jeju	8	1.6

**Table 4:** Consumer shiitake mushroom purchasing behavior.

Purchase Behavior and Related Factors		Frequency (*n*)	Percentage (%)
Annual purchase frequency	1 time	104	20.8
	2 times	121	24.2
	3 times	63	12.6
	4 times	29	5.8
	5 times	51	10.2
	More than 6 times	132	26.4
Average purchase volume per trip	Less than 300 g	184	36.8
	301 g–500 g	145	29.0
	501 g–1 kg	129	25.8
	More than 1 kg	42	8.4
Reasons for purchasing fresh (vs. dried) shiitake	To consume them fresh	231	46.2
	For various culinary uses	158	31.6
	Lower price (compared to dried)	57	11.4
	Better taste (compared to dried)	50	10.0
	Other (e.g., time-saving, convenience)	4	0.8
Information acquisition channels	Family, friends, relatives, neighbors	178	35.6
	YouTube	35	7.0
	TV/radio	54	10.8
	SNS (social media)	14	2.8
	Internet portal	76	15.2
	Campaign/promotional materials	12	2.4
	Seminar/environmental education	5	1.0
	Store/sales staff	113	22.6
	Books, newspapers, magazines	4	0.8
	Other	9	1.8
Purchase location	Local supermarket (non-corporate)	62	12.4
	Corporate supermarket	85	17.0
	Offline hypermarket (large-scale mart)	153	30.6
	Traditional market	97	19.4
	Department store	9	1.8
	Eco-friendly food store	19	3.8
	Online shopping mall	37	7.4
	Offline independent local food market	20	4.0
	Direct transaction from the producer	18	3.6
Reasons for using the purchase location	Good quality	107	21.4
	Low price	105	21.0
	Easy access	171	34.2
	Delivery available	17	3.4
	One-stop shopping (can buy other items)	43	8.6
	Can compare products	16	3.2
	Kind employees and good service	10	2.0
	To buy local specialty products	17	3.4
	Frequent discounts/sales	7	1.4
	Convenient return/refund policy	2	0.4
	Detailed food information available	5	1.0

**Table 5:** Purchasing behavior differences by age and gender.

Category	Item	Age (%)						Gender (%)		
		20s	30s	40s	50s	60s+	*p*-Value	Male	Female	*p*-Value
Annual purchase frequency	Light (1–2 times)	46.4	48.7	41.8	46.0	44.7	0.253	53.6	36.5	0.001
	Medium (3–5 times)	37.7	28.9	25.3	23.0	29.2		24.6	32.5	
	Heavy (more than 6 times)	15.9	22.4	33.0	31.0	26.1		21.8	31.0	
Purchase location	Offline hypermarket (large-scale mart)	26.1	23.7	37.4	29.0	32.3	0.029	28.6	32.5	0.635
	Traditional market	10.1	23.7	19.8	22.0	19.3		21.4	17.5	
	Corporate supermarket	27.5	30.3	13.2	12.0	11.8		16.9	17.1	
Information acquisition channels	Family, friends, relatives, neighbors	29.0	27.6	47.3	42.0	31.7	0.000	37.9	33.3	0.375
	Store/sales staff	8.7	10.5	22.0	23.0	34.2		19.4	25.8	
	YouTube	21.7	10.5	2.2	4.0	3.7		7.7	6.3	

Note: Percentages represent the proportion within each group. *p*-values are derived from the Pearson Chi-square test. Detailed data are provided in Appendix A.

**Table 6:** Considerations for purchasing fresh shiitake mushrooms.

Consideration Factor	Mean	Std. Dev.
Taste	3.920	0.731
Hygiene/safety	4.034	0.750
Regional specialty	3.666	0.820
Origin	4.040	0.792
Convenience of preparation	3.754	0.739
Packaging condition	3.772	0.754
Variety	3.378	0.832
National certification for food safety	3.774	0.854

**Table 7:** Utilities of each level and relative importance of each attribute.

Attributes	Levels	Utilities	Std. Error	Relative Importance (%)
Cap size	<8 cm	−0.013	0.013	16.636
	≥8 cm	0.013	0.013	
Cap color	Dark	0.147	0.013	17.104
	Light	−0.147	0.013	
Origin	Korean	−0.007	0.013	19.848
	Chinese	0.007	0.013	
Price (per 500 g)	KRW 4000 (USD 2.9)	0.092	0.022	46.411
	KRW 7000 (USD 5.1)	−0.073	0.022	
	KRW 10,000 (USD 7.3)	−0.137	0.022	
	KRW 13,000 (USD 9.5)	0.118	0.022	
Constant		4.500	0.013	

Note: Pearson’s *R* = 0.998 (*p* < 0.001); Kendall’s tau = 1.000 (*p* < 0.001).

**Table 8:** Market share prediction based on the Logit model.

ID	Cap Size	Cap Color	Origin	Price (500 g)	Market Share (%)	Rank
1	≥8 cm	Dark	Chinese	KRW 7000 (USD 5.1)	13.5	3
2	≥8 cm	Light	Korean	KRW 13,000 (USD 9.5)	12.0	5
3	≥8 cm	Dark	Korean	KRW 4000 (USD 2.9)	15.7	2
4	<8 cm	Dark	Chinese	KRW 13,000 (USD 9.5)	15.9	1
5	<8 cm	Dark	Korean	KRW 10,000 (USD 7.3)	12.2	4
6	<8 cm	Light	Chinese	KRW 4000 (USD 2.9)	11.6	6
7	<8 cm	Light	Korean	KRW 7000 (USD 5.1)	9.7	7
8	≥8 cm	Light	Chinese	KRW 10,000 (USD 7.3)	9.4	8

## Data Availability

The data presented in this study are available on request from the corresponding author. The data are not publicly available since they are collected for internal research purposes for the National Institute of Forest Science, Republic of Korea.

## Abbreviations

The following abbreviations are used in this manuscript:

BTL	Bradley–Terry–Luce
CBC	Choice-Based Conjoint
DCE	Discrete Choice Experiment
GAP	Good Agricultural Practices
ID	Identifier
IRB	Institutional Review Board
KRW	Korean Won
NIFoS	National Institute of Forest Science
NTFP	Non-Timber Forest Product
ORTHOPLAN	Orthogonal Design Procedure in SPSS
SNS	Social Networking Service
SPSS	Statistical Package for the Social Sciences
TV	Television
USD	United States Dollar

## Appendix A

**Table A1:** Cross-tabulation of demographic characteristics and annual purchase frequency.

Variable	Category	Light User (1–2 Times)		Medium User (3–5 Times)		Heavy User (≥6 Times)		Total		Chi-Square	df	*p*-Value
		*n*	%	*n*	%	*n*	%	*n*	%			
Age	20s	32	46.4	26	37.7	11	15.9	69	100.0	12.502	10	0.253
	30s	37	48.7	22	28.9	17	22.4	76	100.0			
	40s	38	41.8	23	25.3	30	33.0	91	100.0			
	50s	46	46.0	23	23.0	31	31.0	100	100.0			
	60s	72	44.7	47	29.2	42	26.1	161	100.0			
	70s+	0	0.0	2	66.7	1	33.3	3	100.0			
Gender	Male	133	53.6	61	24.6	54	21.8	248	100.0	14.888	2	0.001
	Female	92	36.5	82	32.5	78	31.0	252	100.0			
Income	Less than 3 million KRW	42	56.0	21	28.0	12	16.0	75	100.0	8.33	6	0.215
	3–5 million KRW	73	44.2	44	26.7	48	29.1	165	100.0			
	5–7 million KRW	58	46.4	36	28.8	31	24.8	125	100.0			
	More than 7 million KRW	52	38.5	42	31.1	41	30.4	135	100.0			

Note: Percentages represent the proportion within each group. *p*-values are derived from the Pearson Chi-square test.

**Table A2:** Cross-tabulation of demographic characteristics and primary purchase location.

Variable	Category	Local Super		Corp. Super		Large Mart		Trad. Market		Dept. Store		Eco Store		Online Mall		Local Food		Producer Direct		Total		Chi-Square	df	*p*-Value
		*n*	%	*n*	%	*n*	%	*n*	%	*n*	%	*n*	%	*n*	%	*n*	%	*n*	%	*n*	%			
Age	20s	10	14.5	19	27.5	18	26.1	7	10.1	3	4.3	4	5.8	6	8.7	0	0.0	2	2.9	69	100.0	58.623	40	0.029
	30s	9	11.8	23	30.3	18	23.7	18	23.7	2	2.6	3	3.9	3	3.9	0	0.0	0	0.0	76	100.0			
	40s	11	12.1	12	13.2	34	37.4	18	19.8	0	0.0	2	2.2	5	5.5	6	6.6	3	3.3	91	100.0			
	50s	12	12.0	12	12.0	29	29.0	22	22.0	2	2.0	7	7.0	10	10.0	4	4.0	2	2.0	100	100.0			
	60s	20	12.4	19	11.8	52	32.3	31	19.3	2	1.2	3	1.9	13	8.1	10	6.2	11	6.8	161	100.0			
	70s+	0	0.0	0	0.0	2	66.7	1	33.3	0	0.0	0	0.0	0	0.0	0	0.0	0	0.0	3	100.0			
Gender	Male	30	12.1	42	16.9	71	28.6	53	21.4	6	2.4	7	2.8	22	8.9	8	3.2	9	3.6	248	100.0	6.111	8	0.635
	Female	32	12.7	43	17.1	82	32.5	44	17.5	3	1.2	12	4.8	15	6.0	12	4.8	9	3.6	252	100.0			
Income	<3 mil. KRW	13	17.3	11	14.7	16	21.3	14	18.7	2	2.7	3	4.0	6	8.0	5	6.7	5	6.7	75	100.0	29.405	24	0.205
	3–5 mil. KRW	20	12.1	40	24.2	51	30.9	30	18.2	2	1.2	5	3.0	9	5.5	5	3.0	3	1.8	165	100.0			
	5–7 mil. KRW	12	9.6	22	17.6	41	32.8	25	20.0	1	0.8	3	2.4	12	9.6	3	2.4	6	4.8	125	100.0			
	>7 mil. KRW	17	12.6	12	8.9	45	33.3	28	20.7	4	3.0	8	5.9	10	7.4	7	5.2	4	3.0	135	100.0			

Note: Percentages represent the proportion within each group. *p*-values are derived from the Pearson Chi-square test. Abbreviations: Local Super = Local supermarket (non-corporate); Corp. Super = Corporate supermarket; Large Mart = Offline hypermarket (large-scale mart); Trad. Market = Traditional market; Dept. Store = Department store; Eco Store = Eco-friendly food store; Local Food = Offline independent local food market; Producer Direct = Direct transaction from producer; mil. KRW = million Korean Won.

**Table A3:** Cross-tabulation of demographic characteristics and information acquisition channels.

Variable	Category	Family/ Friends		YouTube		TV/ Radio		SNS		Internet Portal		Camp./ Promo		Seminar/ Edu		Store Staff		Books		Other		Total		Chi-Square	df	*p*-Value
		*n*	%	*n*	%	*n*	%	*n*	%	*n*	%	*n*	%	*n*	%	*n*	%	*n*	%	*n*	%	*n*	%			
Age	20s	20	29.0	15	21.7	7	10.1	7	10.1	8	11.6	2	2.9	3	4.3	6	8.7	0	0.0	1	1.4	69	100.0	128.251	45	0.000
	30s	21	27.6	8	10.5	19	25.0	1	1.3	15	19.7	2	2.6	1	1.3	8	10.5	1	1.3	0	0.0	76	100.0			
	40s	43	47.3	2	2.2	4	4.4	2	2.2	18	19.8	1	1.1	0	0.0	20	22.0	0	0.0	1	1.1	91	100.0			
	50s	42	42.0	4	4.0	9	9.0	2	2.0	14	14.0	2	2.0	1	1.0	23	23.0	1	1.0	2	2.0	100	100.0			
	60s	51	31.7	6	3.7	15	9.3	2	1.2	21	13.0	5	3.1	0	0.0	55	34.2	2	1.2	4	2.5	161	100.0			
	70s+	1	33.3	0	0.0	0	0.0	0	0.0	0	0.0	0	0.0	0	0.0	1	33.3	0	0.0	1	33.3	3	100.0			
Gender	Male	94	37.9	19	7.7	31	12.5	5	2.0	39	15.7	6	2.4	3	1.2	48	19.4	1	0.4	2	0.8	248	100.0	9.704	9	0.375
	Female	84	33.3	16	6.3	23	9.1	9	3.6	37	14.7	6	2.4	2	0.8	65	25.8	3	1.2	7	2.8	252	100.0			
Income	<3 mil. KRW	19	25.3	8	10.7	7	9.3	4	5.3	15	20.0	1	1.3	0	0.0	20	26.7	0	0.0	1	1.3	75	100.0	26.801	27	0.475
	3–5 mil. KRW	70	42.4	10	6.1	16	9.7	3	1.8	21	12.7	5	3.0	2	1.2	36	21.8	0	0.0	2	1.2	165	100.0			
	5–7 mil. KRW	37	29.6	9	7.2	19	15.2	5	4.0	22	17.6	3	2.4	2	1.6	24	19.2	1	0.8	3	2.4	125	100.0			
	>7 mil. KRW	52	38.5	8	5.9	12	8.9	2	1.5	18	13.3	3	2.2	1	0.7	33	24.4	3	2.2	3	2.2	135	100.0			

Note: Percentages represent the proportion within each group. *p*-values are derived from the Pearson Chi-square test. Abbreviations: Camp. = Campaign; Promo = Promotional materials; Edu = Education; mil. KRW = million Korean Won.

## References

[1] Ahmad I., Arif M., Xu M., Zhang J., Ding Y., Lyu F. (2023). Therapeutic Values and Nutraceutical Properties of Shiitake Mushroom (*Lentinula edodes*): A Review. Trends Food Sci. Technol..

[2] Jin G.-E., Um S.-N., Lee D.-H., Bang M.-H., Yang H.-J., Park K.-M. (2009). Analysis of Substances for Chronic Disease Prevention and Immune Enhancement in the Fruiting Body Extracts of Shiitake Mushroom (*Lentinus edodes*). J. Mushrooms.

[3] Meinhold K., Darr D. (2019). The Processing of Non-Timber Forest Products through Small and Medium Enterprises—A Review of Enabling and Constraining Factors. Forests.

[4] Boyapati T., Muthukumarappan K. (2025). Non-Timber Forest Products and the Bioeconomy: Linking Livelihood Security and Biodiversity Conservation (2015–2025 Trends). Front. Sustain. Food Syst..

[5] Kim Y.-D., Kim M.-E., Min K.-T. (2015). The Study on Changes of Consumption Patterns on Oak-Mushrooms: 2006-2014. Korean J. For. Econ..

[6] Min J.-A., Chang Y., Kim J., Jung B.-H. (2024). A Study on Changes in the Consumption Behavior of Non-timber Forest Products in the Gyeonggi, Seoul, Incheon. J. Korean Data Anal. Soc..

[7] Hwang Y. (2007). A Study on the Assessment of Agricultural Product Brands.

[8] Kim D.H., Jeong B.H., Song S.H., Lee H.S. (2024). Estimations of Demand and Supply Model for Fresh Shiitake Mushroom (*Lentinula edodes*). J. Agric. Life Sci..

[9] Rha J.-Y., Lee B., Chun Y., Han S., Ko J. (2020). Factors Influencing Consumer Choice for HMR Using Local Food—Focused on Alphabet Theory. J. East Asian Soc. Diet. Life.

[10] Lizin S., Rousseau S., Kessels R., Meulders M., Pepermans G., Speelman S., Vandebroek M., Van Den Broeck G., Van Loo E.J., Verbeke W. (2022). The State of the Art of Discrete Choice Experiments in Food Research. Food Qual. Prefer..

[11] Jo W.-S. (2024). Analysis of research trends on mushrooms using the report from the Ministry of Agriculture in Korea. J. Mushrooms.

[12] Srisukwatanachai T., Jiang B., Boonkong A., Kassoh F.S., Senawin S. (2025). The Impact of Sensory Perceptions and Country-of-Origin Practices on Consumer Preferences for Rice: A Comparative Study of China and Thailand. Foods.

[13] Jordaan D., Mielmann A., Brits C. (2025). Consumers’ Perceived Value of Pork Meat: A Segmentation on Intrinsic and Extrinsic Cues. Foods.

[14] Go D.-H., Yi S. (2025). Assessing Consumer Valuation of Sustainability Certification in Seafood Products: Insight from a Discrete Choice Experiment of Korean Blue Food Market. Foods.

[15] Green P.E., Krieger A.M., Wind Y. (2001). Thirty Years of Conjoint Analysis: Reflections and Prospects. Interfaces.

[16] Ha C.-S., Lee H.-Y. (2012). The Validity of Conjoint Models with the Attributes and Their Levels Determined by the Respondents. J. Mark. Manag. Res..

[17] Jung B.-H., Kim E.-G., Lee S.-Y. (2003). An Analysis of Korean Consumer and Preferences for Oak-Mushroom. Korean J. For. Econ..

[18] Min K., Koo J.-C. (2020). Chinese Consumer’s Behavior and Preference Attributes in Consumption of Mushrooms. Korean J. For. Econ..

[19] Green P.E., Rao V.R. (1971). Conjoint Measurement-for Quantifying Judgmental Data. J. Mark. Res..

[20] Montgomery D.C. (2017). Design and Analysis of Experiments.

[21] Orme B.K. (2010). Getting Started with Conjoint Analysis: Strategies for Product Design and Pricing Research.

[22] Vriens M. (1994). Solving Marketing Problems with Conjoint Analysis. J. Mark. Manag..

[23] Manalo A.B. (1990). Assessing the Importance of Apple Attributes: An Agricultural Application of Conjoint Analysis. Northeast. J. Agric. Resour. Econ..

[24] Kim K.D. (2015). Consumer Consumption Behavior and Preference of Salted Wild Vegetable: A conjoint analysis with Allium victorialis. J. Korean Soc. For. Sci..

[25] Dijkstra J., Roelen W.A.H., Timmermans H.J.P. Conjoint Measurement in Virtual Environments—A Framework: Conference. Proceedings of the 3rd Design and Decision Support Systems in Architecture and Urban Planning Conference.

[26] Ben-Akiva M., McFadden D., Train K. (2019). Foundations of Stated Preference Elicitation: Consumer Behavior and Choice-Based Conjoint Analysis. Found. Trends^®^ Econom..

[27] Holopainen J., Toppinen A., Lähtinen K., Rekola M. (2017). Forest Certification and Country of Origin: Choice Experiment Analysis of Outdoor Decking Material Selection in e-Commerce Market in Finland. Forests.

[28] Park J.-M., Yang T.-H., Park S.H., Yeo G.-T. (2020). Study on Analysis of Difference in Preference of Stakeholders in Port Gyeongin Using Conjoint Analysis. J. Navig. Port Res..

[29] McFadden D. (1972). Conditional Logit Analysis of Qualitative Choice Behavior.

[30] Beak S., Rhim H., Park M. (2006). A Study on Product Positioning based on Conjoint Analysis in a Competitive Market. J. Korean Oper. Res. Manag. Sci. Soc..

[31] Chung I.-M., Kim Y.-J., Kwon C., Moon H.-S., Han J.-G., Kong W.-S., Kim S.-H. (2021). An Origin Identification Model for Labeling of Shiitake (*Lentinula edodes*). npj Sci. Food.

[32] Louviere J.J., Hensher D.A., Swait J.D. (2000). Stated Choice Methods: Analysis and Applications.

[33] Louviere J.J., Flynn T.N., Carson R.T. (2010). Discrete Choice Experiments Are Not Conjoint Analysis. J. Choice Model..

[34] Schrobback P., Zhang A., Loechel B., Ricketts K., Ingham A. (2023). Food Credence Attributes: A Conceptual Framework of Supply Chain Stakeholders, Their Motives, and Mechanisms to Address Information Asymmetry. Foods.

[35] De Cianni R., Pippinato L., Mancuso T. (2023). A Systematic Review on Drivers Influencing Consumption of Edible Mushrooms and Innovative Mushroom-Containing Products. Appetite.

[36] Zeithaml V.A. (1988). Consumer Perceptions of Price, Quality, and Value: A Means-End Model and Synthesis of Evidence. J. Mark..

[37] Rao A.R., Monroe K.B. (1989). The Effect of Price, Brand Name, and Store Name on Buyers’ Perceptions of Product Quality: An Integrative Review. J. Mark. Res..

[38] Yue C., Tong C. (2009). Organic or Local? Investigating Consumer Preference for Fresh Produce Using a Choice Experiment with Real Economic Incentives. HortScience.

[39] Davidson K.A., Khanal B., Messer K.D. (2024). Are Consumers No Longer Willing to Pay More for Local Foods? A Field Experiment. Agric. Resour. Econ. Rev..

[40] Chakrabarti A., Campbell B.L., Shonkwiler V. (2019). Eliciting Consumer Preference and Willingness to Pay for Mushrooms: A Latent Class Approach. J. Food Distrib. Res..

[41] Owusu R., Anang B.T. (2023). Consumption and Preferences for Mushrooms in Ghana: A Comparison between Regret-Based and Utility-Based Approaches. Food Humanit..

[42] Zhou X., Perez-Cueto F.J., Santos Q.D., Monteleone E., Giboreau A., Appleton K.M., Bjørner T., Bredie W.L., Hartwell H. (2018). A Systematic Review of Behavioural Interventions Promoting Healthy Eating among Older People. Nutrients.

